# The Influence of Acupressure at Extra 1 Acupuncture Point on the Spectral Entropy of the EEG and the LF/HF Ratio of Heart Rate Variability

**DOI:** 10.1093/ecam/nen061

**Published:** 2011-02-17

**Authors:** Young-Chang P. Arai, Takahiro Ushida, Takako Matsubara, Kazuhiro Shimo, Hiroshi Ito, Yuko Sato, Yoshiko Wakao, Toru Komatsu

**Affiliations:** ^1^Multidisciplinary Pain Centre, Aichi Medical University, School of Medicine, 21 Karimata, Nagakutecho, Aichigun, Aichi, 480-1195, Japan; ^2^Department of Anaesthesiology, Aichi Medical University, School of Medicine, 21 Karimata, Nagakutecho, Aichigun, Aichi, 480-1195, Japan

## Abstract

Acupressure applied on the Extra 1 acupuncture point results in sedation, thereby reducing bispectral index (BIS) values. Mental status and hypnotic agents influence the autonomic nervous system. We hypothesized that acupressure at the Extra 1 point would induce sedation and change sympatho-parasympathetic nerve balance. We investigated the effect of acupressure at the Extra 1 point on the EEG spectral entropy values and heart rate variability (HRV). Forty-eight volunteers (24 males and 24 females) were randomly assigned to the control or Extra 1 group. The control group received acupressure at a sham point and the Extra 1 group received acupressure at the Extra 1 point. Acupressure was applied for 5 min. The record of the EEG spectral entropy values and HRV started 5 min before acupressure and stopped 5 min after acupressure. Acupressure significantly reduced the EEG spectral entropy values in both groups, but the values of the Extra 1 group were significantly smaller than those of the control group (*P* < .01). Acupressure significantly decreased the LF/HF ratio of HRV in both groups (*P* < .05). When divided upon gender, although acupressure tended to decrease the LF/HF ratio, the ratio significantly decreased during and after acupressure only in females of the Extra 1 group (*P* < .05). We concluded that acupressure on the Extra 1 point significantly reduced the EEG spectral entropy in both the genders, but affected the LF/HF ratio of HRV only in females.

## 1. Introduction

Pain relief, relaxation, anti-nausea and anti-vomiting and the other effects are provided by acupuncture techniques applied on the traditionally used acupuncture points [1–4]. Although acupressure applied on the Extra 1 acupuncture or Yintang point, results in sedation and thereby reduces bispectral index (BIS) values [5], there are no changes in heart rate and arterial blood pressure observed [6]. Mental status and hypnotic agents are known to influence the autonomic nervous system (ANS) [7–9]. Also, several studies showed that acupuncture and magnitopuncture on the traditional acupuncture point influence the ANS [10, 11]. That is, these procedures could modulate the activities of the sympathetic and parasympathetic nerves.

Frequency domain analysis of heart rate variability (HRV) is a sophisticated noninvasive tool to assess ANS regulation of the heart [11, 12]. Frequency fluctuations in the range of 0.04–0.15 Hz (low frequency, LF) are considered to be markers of sympathetic and parasympathetic nerve activity, and high frequency (HF) fluctuations in the range of 0.15–0.4 Hz are considered markers of parasympathetic nerve activity. Thus, the LF/HF ratio is considered to be an index of sympathetic nerve activity [13, 14].

Recent developments in the analysis of cortical electrical activity lead to the introduction of devices and mathematical algorithms derived from the electroencephalogram (EEG). The ENTROPY index monitor is one of these devices. The entropy algorithm of the ENTROPY is based on two spectral entropy values, namely, state entropy, derived over the frequency range 0.8–32 Hz, and response entropy, derived over the frequency range 0.8–47 Hz [15, 16]. State entropy includes mostly the EEG-dominant spectrum, reflecting primarily the cortical activity of the patients, whereas response entropy includes both the EEG-dominant and EMG-dominant part of the spectrum. A study showed that spectral entropy is a valid indication of the hypnotic effect of anesthetics [15].

We hypothesized that acupressure on the Extra 1 point would induce sedation, thereby changing EEG and autonomic nervous activity. In the present study, we therefore investigated the effect of pressure applied on the Extra 1 point on the spectral entropy of the EEG and the LF/HF ratios of HRV in volunteers.

## 2. Methods

### 2.1. Subjects

After obtaining approval from the local ethics committee, 48 volunteers who were ASA physical status I gave their consent to participate in the present study. No one was taking sedatives, analgesics or other drugs. The volunteers were randomly allocated to two groups of 24 each using computer-generated random numbers. Twelve male and 12 female volunteers were included in each group.  Table 1 shows the demographic data of the two groups.

### 2.2. Group and Administration

Volunteers in the control group received acupressure at a sham point and volunteers in the Extra 1 group received acupressure at the Extra 1 point. All measurements were performed during morning hours. Each volunteer was lying comfortably in an armchair in sitting position and in a quiet environment. ECG electrodes were attached for HRV analysis and the ECG signals were obtained from conventional anesthesia monitor (Hewlett Packard, Model 66s). The data were transferred to an online computer loaded with HRV analysis software (TARAWA/WIN; Suwa Trust, Tokyo, Japan). For real-time analysis, the R-R intervals (RRI) were obtained in the accuracy of 1 ms and analyzed with the “MemCalc" computer program [9, 14]. The two series of power of the RRI (ms2), LF and HF, were calculated. Heart rate (HR) and the LF and HF values and LF/HF ratios of HRV were recorded. The spectral entropy of the EEG was measured with a plug-in Datex-Ohmeda M-Entropy S/5TM module (Datex-Ohmeda Division, Instrumentarium Corp., Helsinki, Finland) [15, 16]. Before sensor application, the skin of the forehead was carefully cleaned with an alcohol swab and allowed to dry. The entropy electrode was positioned, as recommended by the manufacturer, onto the temporal-frontal area of the patient's forehead. Impedances were continuously checked to be <7.5 kOhm. Entropy parameters are calculated from two different frequency ranges. State entropy is computed from 0.8 to 32 Hz, which consists predominantly of EEG. Response entropy includes additional higher frequencies up to 47 Hz, reflecting the inclusion of fast muscle activity from the frontal muscle. The sampling rate was 400 Hz for state entropy and response entropy and the monitored parameters were collected with a laptop computer (Toshiba Satellite, Toshiba Corp., Tokyo, Japan).

**Figure 1:**
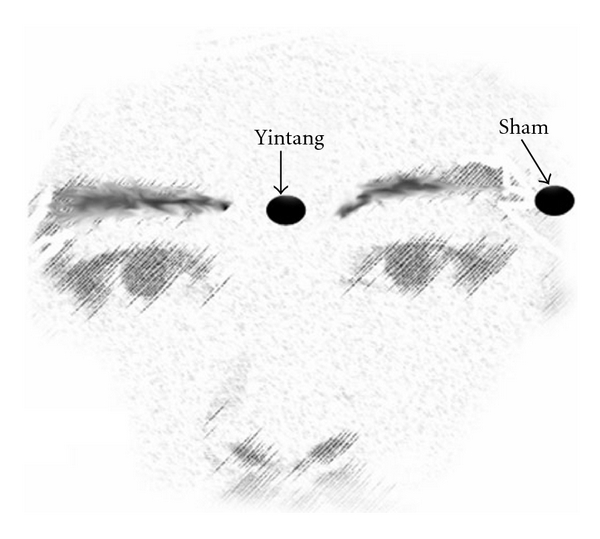
The locations of Extra 1 and sham points.

The Extra 1 acupuncture point is located midway between the medial ends of the two eyebrows at the root of the nose and the sham point is located 2 cm lateral and horizontal from the lateral end of the left eyebrow (Figure 1) [5, 6]. After arrival in the quiet environment, the volunteers were allowed to relax for 10 min. Thereafter, the record of the ECG signals and the spectral EEG entropy started. 5 min later, acupressure was applied by the pulp of the right thumb in a rotary fashion at 20–25 cycles min^−1^ for 5 min by the same investigator [5, 6], depending upon the group allocation. Following release of acupressure, the volunteers were observed for another 5 min. The data of the spectral entropy values, and HR and HRV values 1 (as control, T1), 2.5 (T2) and 5 (T3) min after the beginning of the recording, during pressure application on the Extra 1 point for 1 (T4), 2.5 (T5) and 5 (T6) min, and 1 (T7), 2.5 (T8) and 5 (T9) min after pressure release were sampled for subsequent analysis.

### 2.3. Data Analysis

A pilot study using 20 volunteers showed the mean (SD) of the LF/HF ratio of HRV at T1 and T4 to be 4.0 (3.0) and 1.6 (1.3) in the extra 1 group, and 2.8 (2.5) and 0.9 (1.0) in the sham group, respectively. Thus, the sample size of at least 21 was needed for each group to show a difference of 2.5 (2.0) for the LF/HF ratio within the group, when using ANOVA, with a significance level of 0.05 (**α** = 0.05) and a power of 80% (**β** = 0.20). Data are presented as mean (SD). Friedman test was used to analyze the spectral entropy values and HR and HRV values within group, followed by Student-Newman-Keuls test for multiple comparisons. Inter-group comparison was done with Mann-Whitney test. The volunteers of each group were allocated further according to the gender. The demographic data were analyzed by Kruskal-Wallis test followed by Student-Newman-Keuls test for multiple comparisons. Friedman test was used to analyze the spectral entropy values, and HR and HRV values within group, followed by Student-Newman-Keuls test for multiple comparisons. Inter-group comparison for each gender was done with Mann-Whitney test. *P* < .05 was taken as statistically significant.

## 3. Results

### 3.1. EEG

**Figure 2:**
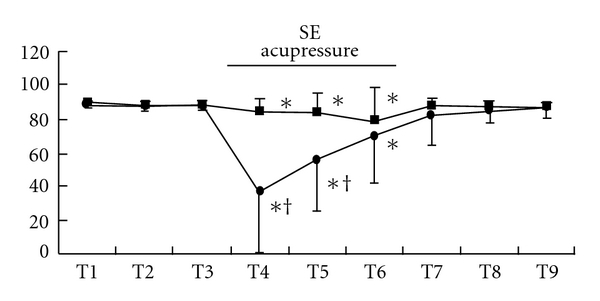
Changes in state entropy value. Data are presented as mean (SD). Filled square, control group; filled circle, Extra 1 group. *Significantly different from control value (*P* < .05). ^†^Significantly different from control group (*P* < .05).

**Figure 3:**
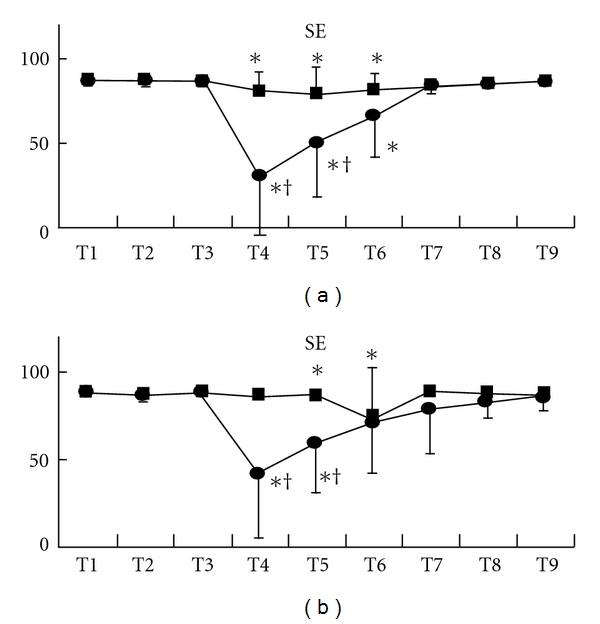
Changes in state entropy value in each gender, males (a) and females (b). Data are presented as mean (SD). Filled square, control group; filled circle, Extra 1 group. *Significantly different from control value (*P* < .05). ^†^Significantly different from control group (*P* < .05).

**Figure 4:**
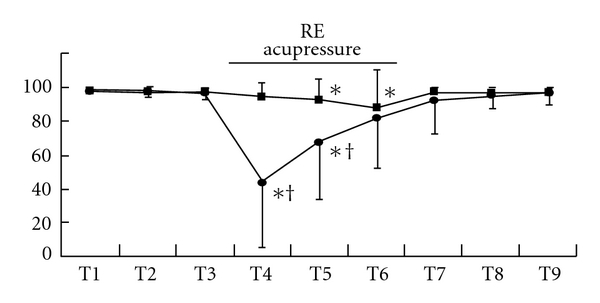
Changes in response entropy value. Data are presented as mean (SD). Filled square, control group; filled circle, Extra 1 group. *Significantly different from control value (*P* < .05). ^†^Significantly different from control group (*P* < .05).

**Figure 5:**
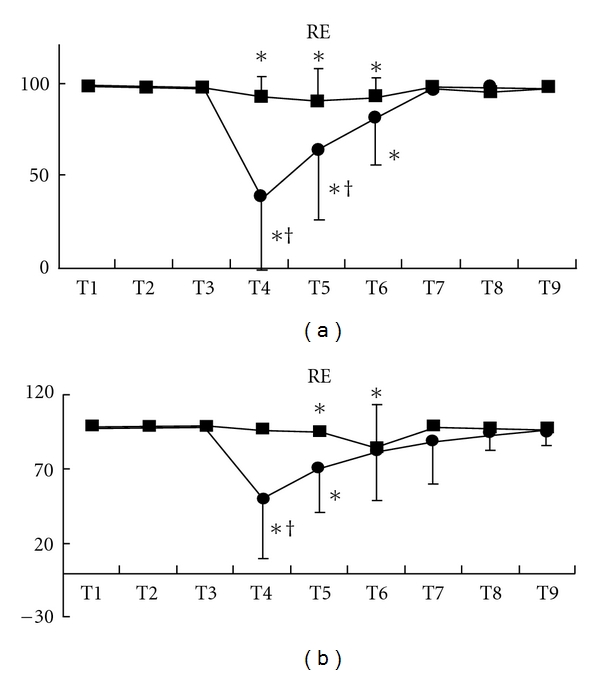
Changes in response entropy value in each gender, males (a) and females (b). Data are presented as mean (SD). Filled square, control group; filled circle, Extra 1 group. *Significantly different from control value (*P* < .05). ^†^Significantly different from control group (*P* < .05).

Although state entropy statistically significantly decreased after the beginning of acupressure in each group, the values of the Extra 1 group were significantly smaller than those of the control group [mean (SD), Extra 1 versus control: T4, 37 (36) versus 84 (9), *P* < .001; T5, 55 (30) versus 83 (12), *P* < .0001] (Figure 2). After acupressure release, state entropy values of these groups were at the control level. These changes were observed in both gender (male, T4, *P* = .0011; T5, *P* = .012: female, T4, *P* = .0002; T5, *P* = .0006) (Figures 3(a) and 3(b)). Response entropy also significantly decreased after the beginning of acupressure and the values of the Extra 1 group were significantly smaller as observed in state entropy [mean (SD), Extra 1 versus control: T4, 44 (39) versus 94 (8), *P* < .0001; T5, 67 (33) versus 92 (12), *P* = .0036] (Figure 4). These tendencies were observed in each gender (male, T4, *P* = .0003; T5, *P* = .0303: female, T4, *P* = .0015) (Figures 5(a) and 5(b)).

### 3.2. HRV

**Figure 6:**
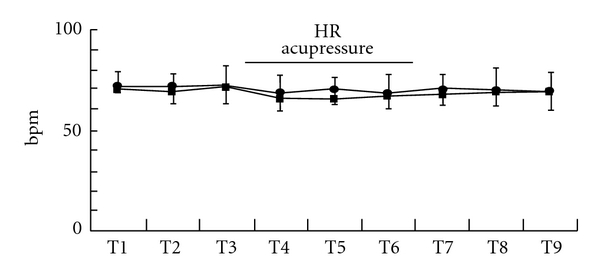
Changes in HR. Data are presented as mean (SD). Filled square, control group; filled circle, Extra 1 group.

**Figure 7:**
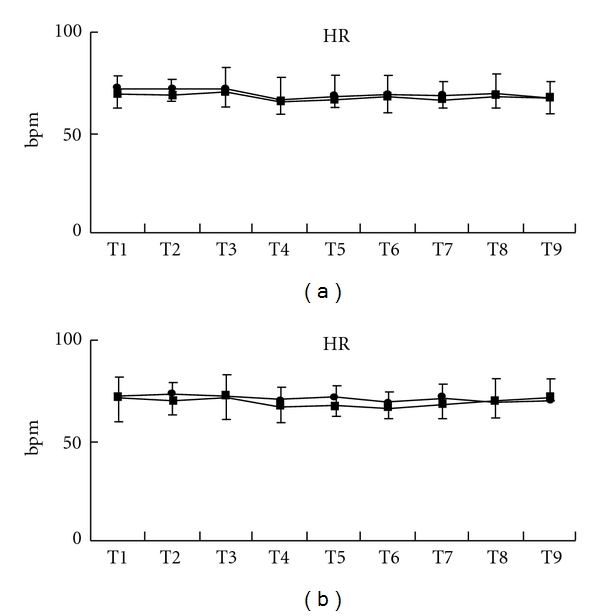
Changes in HR in each gender, males (a) and females (b). Data are presented as mean (SD). Filled square, control group; filled circle, Extra 1 group.

**Figure 8:**
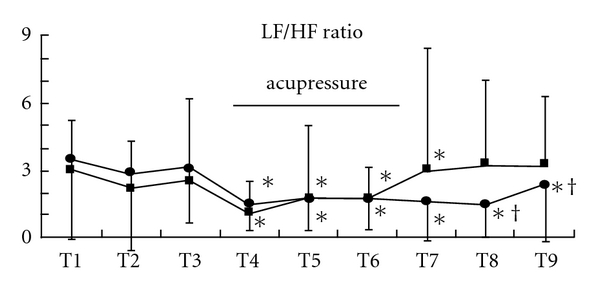
Changes in the LF/HF ratio. Data are presented as mean (SD). Filled square, control group; filled circle, Extra 1 group. *Significantly different from control value (*P* < .05). ^†^Significantly different from control group (*P* < .05).

**Figure 9:**
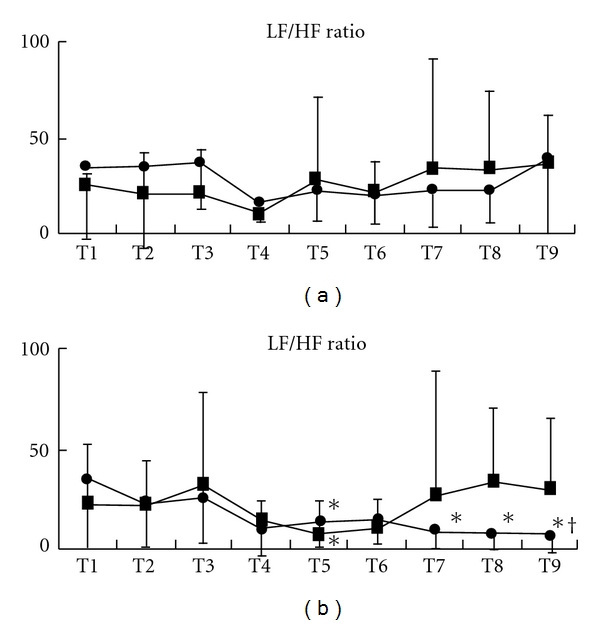
Changes in the LF/HF ratio in each gender, males (a) and females (b). Data are presented as mean (SD). Filled square, control group; filled circle, Extra 1 group. *Significantly different from control value (*P* < .05). ^†^Significantly different from control group (*P* < .05).

Acupressure did not lead to significant changes of HR in both groups (Figures 6, 7(a), and 7(b)). Acupressure *per se* significantly decreased the LF/HF ratio of HRV in both groups [mean (SD), Extra 1, T1 versus T4–9, 3.6 (3.6) versus, 1.5 (1.2), 1.8 (1.5), 1.8 (1.4), 1.6 (1.7), 1.5 (1.5), 2.3 (3.2), *P* < .05; control, T1 versus T4–7, 2.8 (2.0) versus 1.2 (1.3), 1.8 (3.2), 1.6 (1.4), 3.2 (5.3), *P* < .05) (Figure 8). While the LF/HF ratio immediately returned to the control value in the control group after acupressure, the ratio of the Extra 1 group remained a decreased value after acupressure [mean (SD), Extra 1 versus control, T8, 1.5 (1.5) versus 3.5 (3.7), *P* = .05; T9, 2.3 (3.2) versus 3.3 (3.0), *P* = .05). There were no differences in the base line values of both the genders before acupressure application. Furthermore, acupressure significantly decreased the LF/HF ratio of HRV in female volunteers compared with male volunteers in both groups and the LF/HF ratio remained a significantly decreased value after acupressure only in females of the Extra 1 group [mean (SD), Extra 1, T1 versus T4 and 7–9, 3.5 (3.5) versus, 1.3 (1.3), 0.9 (0.8), 0.8 (0.8), 0.7 (0.8), *P* < .05; control, T1 versus T4, 3.0 (3.0) versus 1.4 (1.6), *P* < .05) (Figures 9(a) and 9(b)).

## 4. Discussion

Our results demonstrate that acupressure *per se* significantly reduced two spectral entropy parameters of the EEG, response entropy and state entropy, but acupressure on the Extra 1 point induced greater decreases of response entropy and state entropy, when compared with acupressure on the inappropriate site. Although acupressure did not change HR, acupressure *per se* significantly reduced the LF/HF ratio of HRV. When divided upon gender, although acupressure tended to decrease the LF/HF ratio, the ratio significantly decreased during and after acupressure only in females of the Extra 1 group. The results show that acupressure on the Extra 1 point has a sexually different effect on the autonomic nervous system without an association of changes of the EEG.

Several reports investigated the impact of acupuncture on perioperative managements [2, 3, 17, 18]. Similarly, many investigators tested the effect of acupressure and acupuncture on anxiety, stress and fear, in order to relax the patients and volunteers without contributing to drowsiness or nausea and vomiting [4–6].

Anesthesia depth monitors using the processed EEG have been studied extensively. The BIS monitor is the one most widely studied [19]. The entropy monitor is another recently developed anesthesia depth monitor. It measures the degree of entropy in the EEG. The entropy falls with increasing concentrations of anesthetic [16]. The entropy monitor consists of two outputs: response entropy uses a higher frequency range including EMG; state entropy uses lower frequencies [15, 16]. Several reports showed that acupressure applied on the Extra 1 point significantly reduces the degree of entropy and BIS in the EEG [5, 6, 20–22]. In the present study, similarly, the application of acupressure on the Extra 1 point induced significant decreases of state entropy and response entropy, compared to the application on a sham point. The present results are consistent with the results obtained by BIS monitor. Although we did not check sedating condition, we thus postulate that the sedating effect of acupressure on the Extra 1 point might have led to these decreases of the entropy values.

Clinical and experimental studies showed that differing inputs to somatic nerves have a significant effect on autonomic functions [23, 24]. Acupuncture is also known to affect the autonomic nervous system [25, 26], thereby influencing the cardiovascular system. We showed in the present study that acupressure *per se* tended to reduce the LF/HF ratio of HRV and acupressure on the Extra 1 point especially reduced the LF/HF ratio of female volunteers to a statistically significant level during and after acupressure. That is, the application of acupressure *per se* could influence the ANS and acupressure on a traditional acupuncture point might have a greater effect on the autonomic nervous system in females, because gender is known to influence HRV [26]. Also, there is a possibility that the ANS of female might be more sensitive to acupressure on a traditional acupoint. Furthermore, a study showed gender differences in autonomic functioning during sleep, when using the EEG, with increased sympathetic dominance in males. We thus speculate that sympathetic dominance effect of acupressure-induced sleepy feelings might have prevented a decrease of the sympathetic nerve activity in males [27].

A limitation in the present study is problem of the lack of the control group. In every research, many researchers have argued where the control point was decided. In this study, also, as there are several acupuncture points near the area where we chose to be the sham point, acupressure at the sham point might have induced an unidentified effect in the present study. However, several researchers compared the effect of acupressure at the Extra 1 point with that at the sham point like the present study [5, 6, 10, 11, 28]. Thus, we used the point in the present study. Since electroacupuncture at acupuncture points influence the ANS, acupressure at the sham point might have influenced HRV in the present study. Moreover, in the present study we did not investigate the changes of the same parameters during the same time points with no application of acupressure, and this is one of the limitations of this study. Therefore, we need to do further study in subjects without application of acupressure. Another limitation of our study is that we did not show the effect of the acupressure after the release for a longer period. We need further evaluation of a longer period effect of acupressure on the LF/HF ratio.

In conclusion, acupressure on the Extra 1 point significantly reduced the EEG spectral entropy in both the genders, but affected the power of LF and the LF/HF ratio of HRV only in females.

## Figures and Tables

**Table 1:** Demographic data presented as mean (SD).

	Control			Extra 1		
	Male		Female	Male		Female
		26 (4)			29 (6)	
Age (year)	25 (3)		26 (5)	28 (3)		30 (7)
		164 (12)			164 (9)	
Height (cm)	175 (5)*		153 (6)	172 (4)*		157 (3)
		60 (12)			57 (9)	
Weight (kg)	69 (9)*		51 (6)	64 (7)*		51 (7)

*Significantly different from female volunteers of the group (*P* < .05).
